# Adiponectin as a Biomarker of Osteoporosis in Postmenopausal Women: Controversies

**DOI:** 10.1155/2014/975178

**Published:** 2014-01-23

**Authors:** Anna Lubkowska, Aleksandra Dobek, Jan Mieszkowski, Wojciech Garczynski, Dariusz Chlubek

**Affiliations:** ^1^Department of Functional Diagnostics and Physical Medicine, Faculty of Health Sciences, Pomeranian Medical University in Szczecin, ul. Grudziądzka 31, 70-103 Szczecin, Poland; ^2^Department of Physiology, Faculty of Natural Sciences, Szczecin University, ul. Felczaka 3c, 71-412 Szczecin, Poland; ^3^Institute of Physical Culture, Faculty of Physical Education, Health, and Tourism, Kazimierz Wielki University in Bydgoszcz, ul. Jana Karola Chodkiewicza 30, 85-064 Bydgoszcz, Poland; ^4^Chair and Department of Biochemistry and Medical Chemistry, Pomeranian Medical University, al. Powstańców Wlkp. 72, 70-111 Szczecin, Poland

## Abstract

The literature reports indicating a link between plasma levels of adiponectin and body fat, bone mineral density, sex hormones, and peri- and postmenopausal changes, draw attention to the possible use of adiponectin as an indicator of osteoporotic changes, suggesting that adiponectin may also modulate bone metabolism. In this study, we attempted to analyze the available *in vitro* and *in vivo* results which could verify this hypothesis. Although several studies have shown that adiponectin has an adverse effect on bone mass, mainly by intensifying resorption, this peptide has also been demonstrated to increase the proliferation and differentiation of osteoblasts, inhibit the activity of osteoclasts, and reduce bone resorption. There are still many ambiguities; for example, it can be assumed that concentrations of adiponectin in plasma do not satisfactorily reflect its production by adipose tissue, as well as conflicting *in vitro* and *in vivo* results. It seems that the potential benefit in the treatment of patients with osteoporosis associated with the pharmacological regulation of adiponectin is controversial.

## 1. Introduction

**Figure 1:**
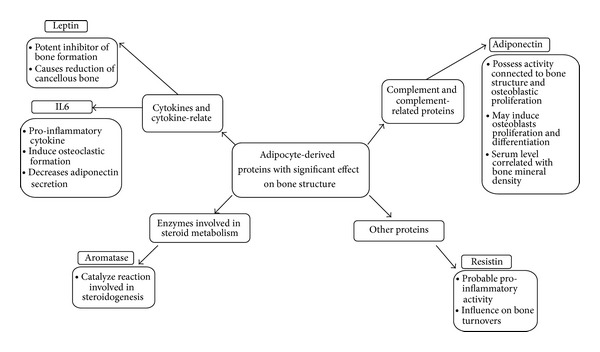
Examples of adipocyte-derived proteins with effect on bone structure.

Although the main role of adipose tissue is energy storage (in the form of free or conducted fatty acids (FFAs)) and thermal protection of the human body, it has been revealed that adipose tissue has an independent endocrine and paracrine activity associated with the production of many bioactive molecules (adipokines) which influence metabolic processes, such as adiponectin (Figure 1) [1].

It has also been shown that metabolites secreted by white adipose tissue (WAT) and brown adipose tissue (BAT) may play an essential role in maintaining normal body weight (regulation of body energy) and that they may participate in maintaining homeostasis, for example, through the prevention of insulin resistance [2, 3]. This may lead to the potential use of these substances as important markers in the prediction of many diseases.

The potential role of adiponectin in diagnostics is associated with protection against atherogenesis, insulin resistance, and obesity and as a possible marker of risk for developing menopausal metabolic syndrome [4–11]. But only a few cross-sectional studies have been performed on the association between serum adiponectin concentrations and bone turnover and bone remodeling markers in humans *in vitro* as well as that clinical studies have shown that serum adiponectin levels are associated with bone mineral density (BMD) and as biochemical markers of bone turnover [12–14]. However, the mechanisms of these associations are unknown and the literature results are still controversial.

On one hand, it has been documented that adiponectin stimulates bone formation and remodeling as well as inhibits bone resorption, suggesting that adiponectin may be a negative regulator of bone mass. On the other hand, most *in vitro* studies demonstrate that adiponectin stimulates the differentiation and mineralization of osteoblasts as well as the expression of osteocalcin, which acts as a hormone regulating glucose metabolism and fat mass [15–18]. The potential influence of adiponectin on osteoblasts and osteoclasts and consequently on bone remodeling can be connected with the interrelationship between the endocrine system and fat metabolism.

Some authors point out that adiponectin could be an independent predictor of BMD and only positively correlates with bone turnover biochemical markers in postmenopausal women, not in premenopausal women [19]. Among the different possible mechanisms, some authors had suggested a link between sex hormones, adiponectin metabolism, production of pro-inflammatory factors, and menopause transition [20–24]. Other reports indicate that total adiponectin levels are significantly lower in obese women compared to nonobese women at the same postmenopausal stage, and it could be inferred that adiponectin is a potential biomarker of osteoporosis in postmenopausal women [25, 26], and this paper attempts to verify this one hypothesis against the available literature data.

## 2. Adiponectin: Structural Characteristics and Function

Adiponectin circulates in 3 different forms in the human body: high molecular weight (18–36 mer), low molecular weight (hexamer), and in a trimeric form. Full-length adiponectin (fAd) requires posttranslational modification for biologic activity (e.g., hydroxylation and glycosylation) and is secreted from adipocytes in three major size classes: trimers (*∼*90 kDa; the basic unit), low-molecular-weight (LMW) hexamers (*∼*180 kDa), and high-molecular-weight (HMW) isoforms consisting of 12-mers to 18-mers (which can exceed 400 kDa) [26, 27]. Although the biological activities of the different forms of adiponectin are poorly understood, they might be specific for distinct receptors and cell types [14].

**Figure 2:**
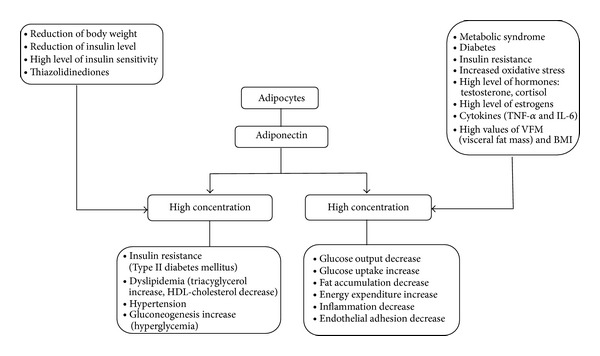
Adiponectin concentration dependent activity.

Adiponectin is an adipocytokine that is highly expressed in human adipose tissue, circulating in serum at concentrations of more than 10 *μ*g/mL [28] (2–15 *μ*g/mL) [29, 30]. It is almost exclusively secreted by adipocytes, especially those in visceral adipose tissue, and to a lesser extent by those in peripheral adipose tissue and bone marrow. Adiponectin concentration-dependent activity is presented in Figure 2. There is a large variation in the levels of adiponectin, which depends on many factors such as age, sex, body mass, and comorbidities (Tables 1 and 2).

The mechanisms of adiponectin activity are not yet fully understood, but a few observations have been well documented.

Adiponectin levels are inversely related to visceral fat and to body mass index [31, 32], and higher levels of adiponectin concentration have been observed in people with anorexia nervosa (up to almost 30%) [29, 30] (Table 2). Secretion of adiponectin may be inhibited by cytokines, such as TNF-*α* and IL-6, and hormones (cortisol and testosterone) whose levels intensively increase in obesity [15]. Nevertheless, decreased levels of adiponectin have been observed in some pathological conditions, increased BMI, type 2 diabetes, and cardiovascular diseases [4, 33, 34]. The levels of adiponectin vary greatly even among subjects with similar BMIs.

The existence of a correlation between hypoglutathionemia and hypoadiponectinemia in subjects with nonalcoholic fatty liver disease (NAFLD) and/or type 2 diabetes mellitus (T2DM) has been proven. Lower adiponectin levels are mostly associated with metabolic syndromes, diabetes, insulin resistance, and dyslipidemia. Yatagai et al. [4] indicated that hypoadiponectinemia was associated with visceral fat accumulation rather than subcutaneous fat deposition in Japanese men with T2DM [4]. Sandhya et al. [5] revealed an association between increased oxidative stress and hypoadiponectinemia in NAFLD subjects with and without T2DM [5]. It is likely that some of the relationships between adiponectin and BMD are due to this relationship with fat mass. It has been proposed that adiponectin may adhere to this tissue as it has been detected in nonadipose tissues such as bone marrow and bone-forming cells. This might lead to the conclusion that adiponectin mediates the association between fat and BMD [12].

## 3. The Interdependence of Adiponectin and the Endocrine System

**Figure 3:**
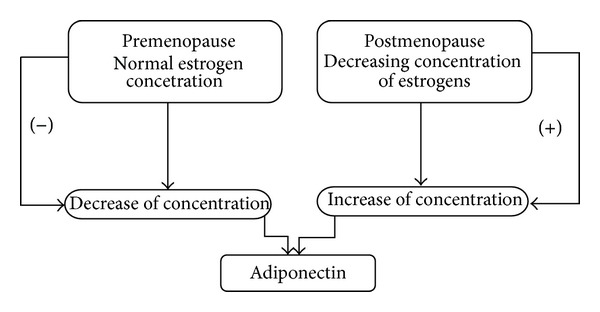
Pre- and postmenopausal adiponectin concentration changes.

Bone mass remodeling and its regulation are under hormonal control. Estrogens, inhibitors of osteoclast-mediated bone resorption, are particularly important. There is also a correlation between estrogen levels and adiponectin concentration [6]. According to Napoli et al., adiponectin serum levels in elderly men are significantly lower than in elderly women [35]. This may be a natural postmenopausal adiponectin increase in women connected to a decreasing concentration of estrogens. Pre- and postmenopausal adiponectin concentration changes are presented in Figure 3.

Some animal models clearly indicate a negative correlation between high estrogen levels and adiponectin production and secretion by adipocytes [36]. This is supported in results of a study by Kunnari et al. who showed that postmenopausal women using estrogen replacement therapy treatment (ERT) had lower adiponectin than a group of women without estrogen supplementation [37]. A similar situation could be observed for testosterone secretion, wherein Page et al. observed that testosterone and its active metabolites could suppress circulating adiponectin concentrations and reduce basal adiponectin concentrations [38]. Unfortunately, the mechanisms of this influence were not clearly explained. There is only a hypothesis that this process may occur as a result of a decrease in fat mass which may result in the decreased secretion of adiponectin by adipocytes. However, such conclusions are not in line with the results of a cross-sectional study by Cnop et al. where high adiponectin levels were connected directly with low abdominal fat mass level [7]. Furthermore, Yang et al. observed that intensive weight loss is associated with increases in serum adiponectin [39]. These results cannot confirm the aforementioned hypothesis that a decrease in fat mass may result in decreased secretion of adiponectin by adipocytes. Therefore, the mechanisms remain unresolved and the only certain fact is that adiponectin shows sex-dependent effects (different in men and women).

## 4. Menopausal Adipose Tissue Metabolism Changes

*Distribution of Adipose Tissue in Pre- and Postmenopausal Women.* The sexually dimorphic distribution of adipose tissue in humans is well known. The main role in the formation of these differences in regulation of adiposity and body fat distribution is played by sex steroids [40]. Puntus et al. observed a positive correlation between BMI values and age among men and women. This association remained strong in premenopausal women but was considerably weaker in postmenopausal women [13]. This supports a theory that premenopausal females tend to have increased lower-body or subcutaneous adiposity, but postmenopausal females tend to have increased visceral or upper-body adiposity, as do men [13].

Menopause has been associated with a decrease in body fat-free mass (FFM) and with a simultaneous increase in body fat mass (FM), in particular abdominal visceral adipose tissue [41]. Other investigations have shown a preferential accumulation of VAT (visceral adipose tissue) in postmenopausal women [41, 42]. Shen et al. and several another studies suggest that a larger volume of VAT with greater age occurs not only in postmenopausal women, but also in the perimenopausal 10 year period [41]. This is in accordance with the observations that the perimenopausal period is associated with changes in the hormonal milieu and the accumulation of visceral fat [41–43].

According to some studies, plasma adiponectin concentrations are negatively correlated with parameters of overall obesity and measures of central obesity in men and women [44–46]. There are reports showing an inverse association between adiponectin concentration and different markers of central obesity: WHR, WTR, BMI, FM, and so forth in women of different ages and body composition [46–49]. Sodi et al. observed positive correlations between body mass index (BMI) and bone mineral density (*r* = 0.44, *P* < 0.001) in a group of postmenopausal women with different body weights, with and without osteoporosis [50]. The authors demonstrated lower adiponectin concentrations amongst the obese women compared to the lean women [50]. A few studies show that adiponectin may influence lipid metabolism [42–48] and perhaps also may affect insulin resistance.

**Figure 4:**
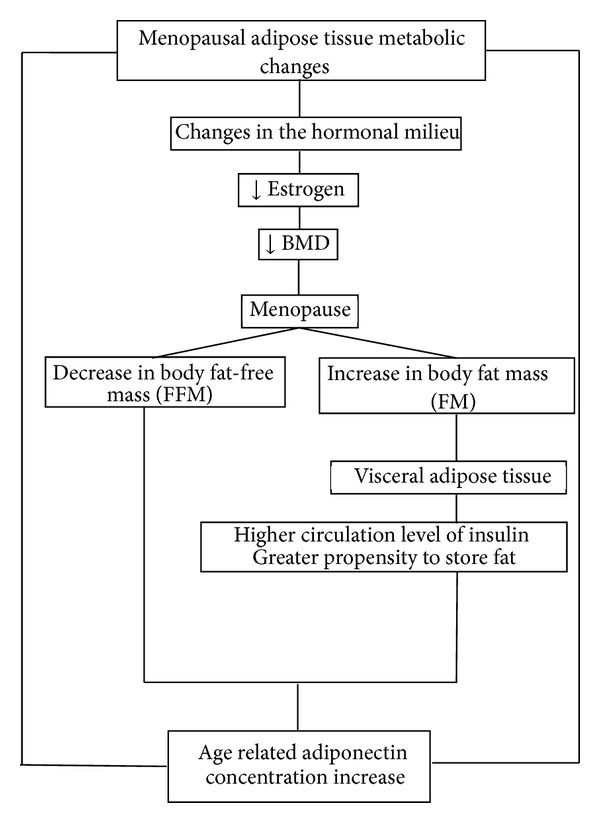
Menopausal adipose tissue metabolic changes.

Menopausal adipose tissue metabolic changes are presented in Figure 4.

## 5. Adiponectin Concentration in Pre- and Postmenopausal Women

Studies about plasma adiponectin concentrations as a function of age in premenopausal and postmenopausal women have reported conflicting results [47].

Circulating adiponectin concentration increases with age in normal-weight, middle-aged, and older women, and its plasma concentration is leptin and age dependent [43, 47]. Some experiments have also shown significantly higher adiponectin levels in postmenopausal than premenopausal women (Table 1). For example, J. Jürimäe and T. Jürimäe showed that middle-aged premenopausal women (40.8 ± 5.7 yr) had lower adiponectin levels than older postmenopausal women (72.2 ± 4.5 yr), with adiponectin concentrations between middle-aged (56.7 ± 3.6 yr) and older postmenopausal women not significantly different [51]. The differences in adiponectin concentrations across the age groups were significant only between pre- and postmenopausal women. Plasma adiponectin concentrations increased with age in healthy, normal-weight, middle-aged, and older women [51]. Furthermore, a complex interaction between specific body composition, bone mineral, cardiorespiratory fitness, metabolic variables, and adiponectin concentration was observed in a relatively homogeneous group of healthy middle-aged and older women [51].

Those conclusions clearly indicate that menopausal status in addition to age appears to have a major influence on adiponectin concentration and body composition in healthy women.

## 6. Sex Hormone Distribution and Adiponectin Concentration during Menopause

Kleerekoper et al. reported that increased aromatization of androgen to estrogens in adipose tissue, lowered sex hormone binding globulin levels with increased free sex steroids, and bone formation induced by increased levels of circulating insulin in obesity may clarify the effects exerted by adipose tissue in the genesis of osteoporosis [20].

Palin et al. indicated that adiponectin, besides its well-known antidiabetic, antiatherogenic, and anti-inflammatory properties, also plays a direct role in reproductive tissues, as evidenced by the presence of adiponectin receptors in the uterus [21]. Furthermore, in women, AdipoR1 and AdipoR2 gene expressions were higher during the mid-secretory phase of the menstrual cycle. This may suggest that adiponectin plays some role in endometrial changes in the preparation for embryoimplantation. In addition, locally produced adiponectin may act as a key neuromodulator for reproductive functions and may also act on the release of gonadotropins. This draws attention to the fact that overweight and obese women have an earlier puberty and a higher risk of developing polycystic ovary syndrome (PCOS), gestational diabetes mellitus (GDM), and preeclampsia. On the other hand, delayed puberty and a higher risk of premature delivery is also associated with underweight women. For this reason, adipose tissue is now recognized as an important factor in the complex equation by which the nutritional status regulates the female reproductive functions [21].

Menopause results in many changes in the metabolites associated with adipose tissues (Figure 4). This change interacts with the hormonal system. Matsui et al. described the association of adipocytokines and ghrelin among obese and nonobese women studied at each of the three menopausal stages [60]. They showed that leptin levels increased across menopause in nonobese women; this change was independently associated with FSH level. Resistin levels were high in premenopause and dropped markedly in perimenopause to levels that were sustained postmenopause. Average adiponectin levels were lower during the perimenopausal stage relative to pre- or postmenopausal stages in both obese and non-obese women. Furthermore, the change in FSH levels was positively associated with a change in adiponectin concentrations. However, the changes in E2, T, and SHBG concentrations were not predictive of adiponectin concentrations. In addition, adiponectin levels were found to be significantly lower during perimenopause compared to both pre- and postmenopause stages [60].

We have found two studies [22, 23] which indicate a significant inverse correlation between adiponectin and estradiol in healthy postmenopausal women, confirmed even after adjustment for age and BMI. In addition, Siemińska et al. indicated that increased levels of free testosterone and low sex hormone-binding globulin (SHBG) in postmenopausal women were associated with decreased levels of adiponectin [24]. The clinical relevance of adipocytokines during menopause is yet to be defined. It seems that adipocytokines may be a link connecting postmenopasual hormonal changes and greater levels of visceral fat [24].

The action of adiponectin on bone in women may be influenced by estrogen. Wang et al. conducted research on the effects of 17*β*-estradiol (E2) on adiponectin-regulated OPG and RANKL expression in human osteoblasts. Through blocking the activation of adiponectin-induced p38 MAPK, E2 suppressed adiponectin-regulated OPG/RANKL expression and then inhibited osteoclastogenesis [25]. These facts clearly indicate that the free testosterone concentration, similar to estrogen levels, negatively correlates with adiponectin serum concentration. Özkurt et al. suggest that new hormonal markers, including adiponectin, may be used as predictors for the rate of bone loss and osteoporotic fracture risk in postmenopausal women, but their prediction rate is only useful in sex hormone-dependent osteoporosis [26].

## 7. Adiponectin Influence on Osteoblastogenesis

Osteoblasts and marrow adipocytes originate from a common mesenchymal progenitor. Research [6] on stem cells has clearly shown that differentiations of bone marrow stem cells into fatty cell lines or bone cell lines are not mutually exclusive. The phenotype of the cells depends on diverse ligands of PPAR*γ*2 (Peroxisome Proliferator-Activated Receptor-*γ*2). This receptor regulates distinct pathways which may lead to full or partial expression of the adipocyte phenotype cell, suppression of osteoblast differentiation, or both. This correlation is very important for the correct understanding of skeletal system metabolism, as there is evidence that marrow fat increases with age in humans in whom osteoblast production is observed [6].

The bone loss during aging in both males and females is due in part to a reciprocal increase in the development of adipocytes and a decrease in osteoblast differentiation. Adiponectin hormone is produced by differentiated adipocytes and its concentration has been observed [25] in a relatively large amount in human serum. It seems essential for osteoblastogenesis that adiponectin and its receptors (AdipoR1 and AdipoR2) are present in bone-forming cells (osteoblasts and osteoclasts) [14, 16, 61]. The presence of adiponectin receptors is different and depends on the localization; AdipoR1 receptor is mostly expressed in the muscles, whereas AdipoR2 is present in the liver. Stimulation of those receptors by adiponectin resulted in increased AMP-activated protein kinase (AMP kinase) and PPAR*α* ligand activity, as well as fatty acid oxidation and glucose uptake in the liver and skeletal muscles [62]. A decrease in the number of AdipoR receptors is the main adiponectin sensitivity influence regulating factor, and it may induce diminution of adiponectin effects (e.g., reduction in tissue insulin sensitivity observed during T2D) [30, 63, 64].

**Figure 5:**
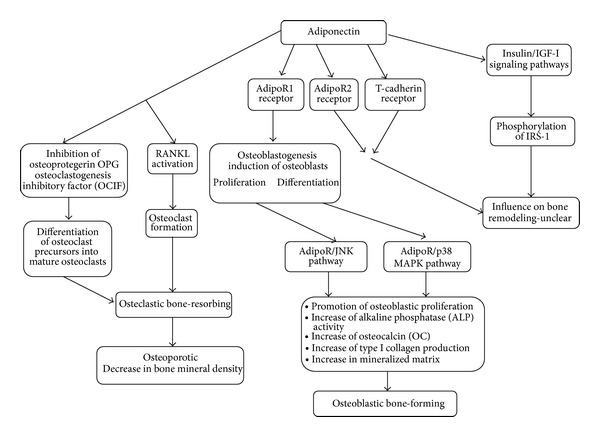
The impact of adiponectin on bone remodeling.

Adiponectin has been observed as a potential factor that may induce osteoblasts proliferation and differentiation. Proliferation activity is mediated through the AdipoR/JNK pathway, and the differentiation activity is directly correlated through the AdipoR/p38 MAPK pathway (Figure 2) [17, 18, 60, 62]. The activity of adiponectin can directly target human osteoblasts. Only adiponectin subtype AdipoR1 receptor activity is correlated with the production of alkaline phosphatase, osteocalcin, while I-collagen and many other bone active factors are correlated with bone density mineralization. This indicates that adiponectin osteoblastic proliferation and differentiation activity take place through AdipoR1. The effect of this activity is the promotion of osteoblastic proliferation in a dose and time dependent increase in alkaline phosphatase (ALP) activity, osteocalcin (OC) and type I collagen production, and an increase in mineralized matrix. This can lead to a conclusion that high adiponectin levels enhance bone mineral density and osteoblast differentiation [65]. These processes are summarized in Figure 5.

Both *in vivo* and *in vitro* research have demonstrated the stimulating effect of adiponectin on the proliferation of osteoblasts with a simultaneous inhibitory effect on osteoclasts [15–18, 66, 67]. For example, it enhanced the expression of alkaline phosphatase mRNA in osteoblastic MC3T3-E1 cells and increased the mineralization of cells. Moreover, the inhibitory effect of adiponectin has been shown to influence the differentiation of mouse bone marrow macrophages and proliferation of osteoclasts [15, 66]. Importantly, Kanazawa et al. have shown that osteoblastic MC3T3-E1 cells expressed AdipoR1, but not AdipoR2 [17]. The inhibition of the expression of AdipoR1 by siRNA inhibited the differentiation and mineralization of cells, and therefore it is believed that these processes are promoted by affecting AdipoR1 without AdipoR2. An example of research confirming an increase in bone mass, accompanied by a reduction in the number of osteoclasts and plasma levels of NTX (a marker of bone resorption), is a paper by Oshima et al. [66] which shows that an increase in adiponectin inhibits M-CSF and RANKL which is induced by bone marrow macrophage differentiation in mice and human CD14—positive mononuclear cells into osteoclasts. The authors also demonstrated the inhibitory effect of adiponectin on the resorptive activity of osteoclasts in bone tissue [66], which is denied, however, by later results presented by Williams et al. [15]. Mitsui et al. [18] present similar results to Oshima et al. in their investigation of the endocrine effects of adiponectin on bone metabolism using a 12-week-old male transgenic mice (Ad-Tg) with significant hyperadiponectinemia over expressing human full-length adiponectin in the liver [66]. They found a significant increase in the levels of osteocalcin in the absence of increased levels of RANKL, osteoprotegerin, and TRAP5b. They also observed a significant increase in bone mass and an increase in bone formation processes. Parameters of bone resorption did not differ between the Ad-Tg mice and the control group [18]. The study poses a conclusion that hyperadiponectinemia exacerbates the processes of bone formation by inhibiting the activation of osteoclasts and osteoblastogenesis [15, 18, 66, 67]. Opposite results were obtained by Shinoda et al. [14] who showed no abnormalities in bone turnover in mice with adiponectin-deficiency and adiponectin over expression in the liver. The effect of adiponectin on bone formation did differ between experimental systems, that is, between *in vivo* and *in vitro*, and between gain-of-function and loss-of-function.

To date, researchers suggest three potential pathways of adiponectin activity in bone: (i) positive action through autocrine/paracrine pathways to locally produced adiponectin, (ii) negative action through the direct pathway by circulating adiponectin, and (iii) a positive action through the indirect pathway by circulating adiponectin via the enhancement of insulin signaling. It has also been demonstrated that blockage of AdipoR1 expression by transfecting siRNA inhibits the differentiation and mineralization of MC3T3-E1 cells. This phenomenon is explained by disorders of the paracrine and autocrine systems as well as the endocrine pathway by siRNA transfection, which in turn leads to the conclusion of the possible impact of cytokines in the activation of osteoblasts in the bone marrow microenvironment [17].

Different adiponectin pathways (hormonal, paracrine, or autocrine) may present some explanation for the lack of visible changes in bone tissue in research by Shinoda et al. [14]. In addition to direct and negative impacts on osteoprogenitor cells, the circulating adiponectin had a beneficial effect on bone remodeling via insulin. It is suggested that the insulin/IGF-I signaling pathways might be alternative candidates for mediating the stimulatory effects of adiponectin on osteoblasts. This was explained by the activating effect of adiponectin on intracellular insulin signaling through phosphorylation of IRS-1 and the serine/threonine protein kinase (Akt), the main downstream molecules of insulin. It has also been shown that increased levels of adiponectin enhance insulin action in its target organs [14]. Given the differences in the aforementioned results, Williams et al. concluded that it is not possible to produce a single model of adiponectin effects on isolated bone cells that can reconcile the disparate findings of all these studies [15]. Thus, differences in the number and activity of osteoblast-like cells in bone marrow cultures could account for some of the inconsistencies between studies, while variation in adipocyte numbers in primary bone marrow cultures could cause variable effects due to local production of adiponectin [15].

In view of the conflicting results of *in vitro* and *in vivo* research, potential benefits in the treatment of patients with osteoporosis resulting from the pharmacological regulation of adiponectin have become controversial and require further research to clarify the effect of different levels and forms of adiponectin on BMD.

The controversy in the interpretation of the effect of adiponectin on bone remodeling has been widened by the results of clinical studies. In contrast to findings in cell culture and animal studies [18, 66], clinical studies demonstrate that circulating adiponectin concentrations are consistently inversely related to BMD (bone mineral density) [12, 28, 35, 52–54, 57, 59, 68–71]. For example, Jürimäe et al. observed that circulating adiponectin appears to exert an independent effect on BMD in perimenopausal women and may represent a link between adipose tissue and bone mineral density [49]. Similarly, J. Jürimäe and T. Jürimäe found significantly higher levels of adiponectin in postmenopausal women compared to premenopausal women, where the relationship between adiponectin concentration and measured bone mineral values was controlled by total FM and insulin resistance in addition to age and menopausal status [51]. Subsequent studies by the same authors [69] confirmed the relationship, showing a negative correlation between plasma adiponectin concentration with total and areal BMD. In addition, they found no significant effect of body composition and hormonal and insulin resistance on BMD. They found that adiponectin is an independent predictor of BMD, while its independent contribution to the interindividual variance in measured values is only modest [69]. Similar results were obtained by Misra et al. who compared women with anorexia nervosa (AN) with women of normal weight [59]. Adolescent girls with AN were at risk of low bone mineral density (BMD), which is associated with hypogonadism, low IGF-I, high cortisol and peptide YY (PYY) levels, and decreased lean mass. Interestingly, they showed increased levels of adiponectin in girls with AN (Table 2). The observed increase in adiponectin contributed significantly to the variability of bone density [59]. A negative correlation between adiponectin and BMD has also been observed in healthy men and women [38, 58].

As previously mentioned [71], an increase in adiponectin activates RANKL and inhibits OPG separation. OPG acts as a soluble decoy receptor for RANK, and by preventing RANKL from binding to RANK, OPG prevents osteoclast activation and decreases apoptosis. This results in an increased osteoclast activity and a reduction in BMD. The research showed no association between adiponectin and OPG and markers of bone turnover, even though researchers have shown that adiponectin is independently associated with BMD in both healthy and AN girls. It is not clear whether this is OPG-mediated [71]. Carrasco et al., who studied morbidly obese women, showed an increase in adiponectin with a corresponding decrease in BMD at 6 and 12 months after gastric bypass surgery, which was associated with a significant weight loss [58]. These results indicated that the mere loss of fat mass increases adiponectin levels and results in a significant loss of BMD (Table 2). However, they emphasized that some metabolic mediators, for example, an adiponectin secretion increase, may have had an independent effect on BMD [58]. The key role of adiponectin in this process is undeniable, though still not fully understood. It seems that the observed dependence between adiponectin and BMD was more important in women after menopause than before menopause, suggesting an important role for estrogen in regulating the effects of adiponectin on BMD (Table 1).

Increased levels of adiponectin have been associated with an increase in the markers of bone turnover, as evidenced by the results of *in vitro* and *in vivo* studies by Kanazawa et al. who showed that total-adiponectin was positively correlated with serum osteocalcin in the presence of vertebral fractures [72]. Some results of reviews and meta-analyses reveal that high serum adiponectin level may be connected with low levels of lumbar BMD [28, 35, 68]. Given that it is speculated that the risk of osteoporosis is increased in people with elevated levels of adiponectin. Therefore, most scientists include high serum levels of adiponectin as a negative factor for bone mass density and osteoporosis [35, 68]. In addition, all sorts of attempts at pharmacological manipulation of the adiponectin pathway designed to raise adiponectin levels in the treatment of obesity-related diseases or reducing insulin resistance in type 2 diabetes may have important negative consequences [54].

On the other hand, there are also reports demonstrating the absence of a correlation between adiponectin and BMD. Three of them [50, 73, 74] concerned young women before menopause while one [74] analyzed the levels of adiponectin in perimenopausal women and one [36] in men. For example, Kontogianni et al. reported an increase in adiponectin levels in postmenopausal women compared with premenopausal women, although they found no significant associations between the levels of circulating adiponectin and BMD assessed at several skeletal sites [74]. Similarly, another study [36] found no link between adiponectin levels and BMD in men. Some interesting and very detailed relationships between adiponectin levels and the level of bone turnover markers have been shown by Sodi et al. where they found no significant difference between the levels of adiponectin in postmenopausal women with or without osteoporosis, which may indicate a lack of effect of adiponectin on osteoporotic changes [50].

However, at this point, it should be remembered that adiponectin does not circulate as a monomer but rather the main circulating forms in human plasma are a 180 KDa low molecular weight (LMW) hexamer and a *∼*360 KDa high molecular weight (HMW) multimer [27, 72]. At present, it remains unclear which form of circulating adiponectin may have a role in bone metabolism although some studies suggest that total adiponectin may have a more potent effect on bone [72]. Researchers [50] did not find an association between total- or HMW-adiponectin and osteoprotegerin (OPG), but instead found a significant and negative correlation between HMW/total adiponectin ratio and OPG. No association was found between total-, HMW-adiponectin, and the HMW/total-adiponectin ratio with either the marker of bone formation, P1NP, or the marker of bone resorption, *β*-CTX. A strong association was found between P1NP and *β*-CTX, which suggests intact coupling between bone resorption and bone remodeling. A sharp increase in the concentration of HMW/total-adiponectin with a simultaneous decline in OPG may indicate that adiponectin lowers the level of osteoclastogenesis inhibitor (OPG), indirectly stimulating bone resorption processes, which explains the reduction in BMD and increased levels of adiponectin concentrations observed in many clinical experiments [50].

In addition, adiponectin has a direct effect on osteoblasts through receptors AdipoR1 and/or AdipoR2. Adiponectin receptors have also been shown to be expressed on osteoclasts and thus, directly or indirectly, may be involved in bone resorption activation [71]. It can be assumed that the outcome of clinical trials may also depend on the differences between adiponectin isoforms. It is worth quoting the study by Richards et al. in which the authors demonstrated a relationship between adiponectin and markers of bone turnover [54]. An increase in adiponectin was associated with increased levels of osteocalcin, which suggests an important role of adiponectin in osteoclastogenesis [54]. In addition, other studies have demonstrated the presence of adiponectin receptors and transcription, translation, and secretion of the adiponectin protein in human bone-forming osteoblasts. Furthermore, the introduction of recombinant adiponectin to human osteoblasts resulted in the stimulation of the formation of osteoblasts and RANK osteoclasts. Thus, the effect of adiponectin on bone metabolism may be due to the promotion of the RANKL pathway [54, 65].

**Figure 6:**
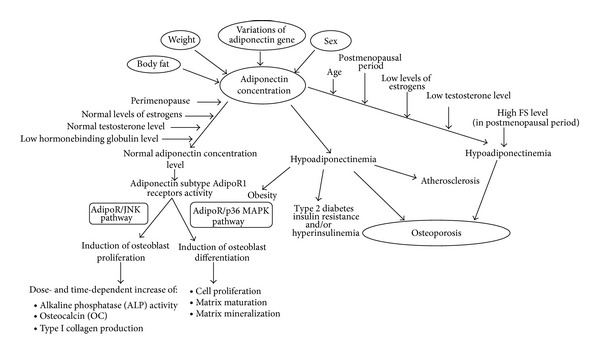
Connection between adiponectin concentration and osteoporosis.

These results show a significant need for a better understanding of adiponectin activity and its influence on bones and the skeletal system. This information could bring information on the possible role of adiponectin in osteoblastic differentiation-dependent diseases. In the first instance, however, we need to establish the actual impact of hormones generated by adipose tissue on bone mass [73]. The interrelationship between adiponectin concentration and osteoporosis is presented in Figure 6.

## 8. Conclusions

It has previously been suggested that the significant correlation between adiponectin level and age is the result of changes in body composition, and it may be associated with physiological hormone changes during the post-menopausal period. Therefore, more accurate clarification is needed of the relationships between biochemical factors and bone structure to reduce the risk of osteoporosis and associated threats. Specific correlations between adiponectin and other biochemical parameters during osteoporosis should give useful information and determine the role of adiponectin use and manipulation in the treatment of osteoporosis, specifically for the cases where osteoporosis is associated with obesity and metabolic syndromes.

In light of the current knowledge, adiponectin cannot be used as an unambiguous predictive marker for osteoporotic fracture risk. Serum adiponectin negatively correlates with increased weight and BMI. However, serum adiponectin does not directly correlate with bone mass and osteoporotic fracture risk. Only age, estrogen level, and leptin concentration seem to be significantly independent predictors of adiponectin levels in the human body. The only indisputable facts are that adiponectin serum level is positively age-dependent, and that BMD during aging is decreasing; the relationship between these two phenomena is still unknown. To sum up, in view of the conflicting results of *in vitro* and *in vivo* studies, potential benefits in the treatment of patients with osteoporosis associated with the pharmacological regulation of adiponectin remain controversial.

## Figures and Tables

**Table 1:** Concentrations of adiponectin and bone mineral density in men and women.

Characteristics of the respondents			Mean ± SD or median (range)						Author
			Age (years)	BMI (kg/m^2^)	Fat-free mass (kg)	Spine BMD (g/cm^2^)	Total BMD (g/cm^2^)	Adiponectin (*µ*g/mL)	
Male	Hemodialysis patients	*n* = 114	61.0 ± 11.1	22.0 ± 3.1	21.5 ± 7.1 (%)	1.019 ± 0.168	—	17.7 (3.1–71.5)	Okuno et al. (2012) [52]
	nondiabetic	*n* = 1164	48.6 ± 14.0	27.1 ± 3.9	—	1.18 ± 1.94	1.21 ± 0.10	8.0 ± 4.1	Zillikens et al. (2010) [53]

Female	Total (pre- and postmenopausal)	*n* = 1467 adult	47.7 ± 14.2	26.4 ± 4.7	—	1.12 ± 0.16	1.10 ± 0.95	12.3 ± 5.8	Zillikens et al. (2010) [53]
		*n* = 153 adult	57.8 ± 13.7	27.6 ± 2.4	43.3 ± 4.0	1.18 ± 0.21	1.18 ± 0.12	12.2 ± 6.3	J. Jürimäe and T. Jürimäe (2007) [51]
		*n* = 1735 adult	50.0 ± 13.0	25.5 ± 4.7	23.06 ± 8.5	1.00 ± 0.1	—	8.3 (3.9)	Richards et al. (2007) [54]
	Premenopausal	*n* = 98 adult	45.2 ± 4.3	29.9 ± 6.2	45.0 ± 4.6	1.24 ± 0.17	1.23 ± 0.12	12.0 ± 4.7	J. Jürimäe and T. Jürimäe (2007) [51]
		middle-age *n* = 42	40.8 ± 5.7	25.9 ± 2.8	45.0 ± 4.8	1.35 ± 0.18	1.26 ± 0.11	8.4 ± 3.2	J. Jürimäe and T. Jürimäe (2007) [51]
		*n* = 25 adult	47.80 ± 3.14	30.01 ± 5.22	42.90 ± 4.92	1.23 ± 0.16	—	7.9 ± 5.81	Kontogianni et al. (2004) [55]
		*n* = 105 adolescents	15.4 ± 1.9	23.1 ± 4.0	24.7 ± 9.3	0.940 ± 0.104	—	30.79 ± 14.48	Huang et al. (2004) [56]
	Postmenopausal non-diabetic (with hip fracture) *n* = 105	nonosteoporosis	58.4 ± 8.2	31.2 ± 5.9	—	0.93 ± 0.11	—	6.33 ± 0.51	Özkurt (2009) [26]
		osteoporosis	68.4 ± 8.0	25.5 ± 9.9	—	0.77 ± 0.2	—	6.99 ± 0.5	
		Total	63.4 ± 8.1	28.5 ± 7.9	—		—	6.66 ± 0.45	
	Postmenopausal	*n* = 84	52.5	29.4	—	0.889	—	13.25	Ağbaht et al. (2009) [12]
		middle-age *n* = 49	56.7 ± 3.6	28.3 ± 2.6	42.5 ± 3.8	1.15 ± 0.16*	1.17 ± 0.09	12.0 ± 5.1	J. Jürimäe and T. Jürimäe (2007) [51]
		older *n* = 62	72.2 ± 4.5	28.5 ± 2.0	42.6 ± 3.0	1.05 ± 0.17	1.12 ± 0.12	15.3 ± 7.3	
		*n* = 55	54.47 ± 5.36	28.89 ± 4.19	40.36 ± 4.56	1.13 ± 0.18	—	11.94 ± 7.00	Kontogianni et al. (2004) [55]

Male & Female (nondiabetic)	Nonosteoporotic	*n* = 120	39.7 ± 10.4	28.8 ± 4.4	—	−0.63 ± 0.43	—	8.03 ± 4.2	Mohiti-Ardekani et al. (2013) [57]
	Osteoporotic	*n* = 81	54.4 ± 15.5	28.2 ± 4.6	—	−3.25 ± 1.1	—	10.7 ± 4.5	

**Table 2:** Summary of concentrations of adiponectin and bone mineral density in patients with morbidly obese women before and after gastric bypass surgery (GBP) and adolescent girls with anorexia nervosa or healthy adolescents.

Characteristics of the respondents	Mean ± SD or median (range)						Author
	Age (years)	BMI (kg/m^2^)	Fat-free mass (kg)	Spine BMD (g/cm^2^)	Total BMD ± SD (g/cm^2^)	Adiponectin (*µ*g/mL)	
Morbidly obese women before gastric bypass surgery (GBP)	37.7 ± 9.6	45.0 ± 4.3	Body fat (%) 47.8 ± 5.1Trunk body fat (%) 45.4 ± 4.8	1.49 ± 0.11	1.23 ± 0.05	—	Carrasco et al. (2009) [58]
Morbidly obese patients after gastric bypass surgery (GBP)							
Baseline		45.0 ± 4.3	58.9 ± 5.8	1.49 ± 0.11	1.23 ± 0.53	11.4 ± 4.3	
6 month		32.5 ± 3.9	48.2 ± 3.8	1.42 ± 0.14	1.21 ± 0.64	15.7 ± 4.8	
12 month		29.5 ± 3.9	48.2 ± 3.6	1.38 ± 0.14	1.19 ± 0.63	19.8 ± 6.6	

Adolescent girls with anorexia nervosa (AN) and healthy adolescents (0, 30, and 60 min after ingestion of a 100 g oral glucose load)							Misra et al. (2007) [59]
AN							
0	—	16.7 ± 1.3	8.9 ± 3.0	−1.20 ± 0.77	—	13.3 ± 6.1	
30						12.5 ± 8.2	
60						11.2 ± 5.4	
Healthy adolescents							
0	—	21.8 ± 3.4	17.9 ± 5.7	−0.34 ± 0.98	—	11.9 ± 7.8	
30						9.8 ± 2.9	
60						8.7 ± 2.8	
